# Predicting wire electrical discharge machined surface roughness of C355/silicon nitride/graphene hybrid nanocomposites using simulation, statistical and machine learning techniques

**DOI:** 10.1038/s41598-026-41376-8

**Published:** 2026-02-27

**Authors:** Suresh Vellingiri, Ravi Kumar Tata, Sumalatha Manne, Arul Natarajan, Sikiru O. Ismail, Arundeep Murugan, Prabhu Paulraj, Senthil Kumar Jeyaramalingam

**Affiliations:** 1 Department of Mechanical Engineering, KIT-Kalaignarkarunanidhi Institute of Technology (Autonomous), Coimbatore, 641 402 Tamil Nadu India; 2 Department of Computer Science and Engineering, Koneru Lakshmaiah Education Foundation, Vaddeswaram, Andhra Pradesh India; 3 Department of Humanities and Sciences, Marri Laxman Reddy Institute of Technology, Hyderabad, Telangana India; 4 Department of Mechatronics Engineering, Sona College of Technology, Salem, 636 005 Tamilnadu India; 5 Centre for Engineering Research, School of Physics, Engineering and Computer Science, University of Hertfordshire, Hatfield, Hertfordshire, AL10 9AB United Kingdom; 6 Department of Mechanical Engineering, Debre Markos University, Debre Markos, Ethiopia; 7 Department of Computer Science and Engineering, KIT- Kalaignarkarunanidhi Institute of Technology (Autonomous), Coimbatore, 641 402 Tamil Nadu India

**Keywords:** Hybrid nanocomposite, WEDM, Simulation, RSM, SVR, ANN, ML techniques, Engineering, Materials science, Nanoscience and technology

## Abstract

This study employed machine learning (ML) and optimization approaches, with support vector regression (SVR), artificial neural networks (ANNs) simulations and response surface methodology to study surface roughness of wire electrical discharge machined/machining of (WEDM) aluminum alloy C355 hybrid composite samples. The samples were strengthened by silicon nitride (Si₃N₄) and graphene nanoparticles (GNPs). The composites surface roughness was investigated using real-time WEDM experiments conducted with varied control settings, including servo-voltage, maximum current, wire feed rate and on/off pulses. The grid-based search approach was used to modify the support vector machine variables, and the layers (input-hidden-output) of the ANN architectural design were achieved. The correlation coefficient and mean absolute percentage error (MAPE) were used to assess the generated models’ prediction ability. SVR outperformed ANN (*R* = 0.991350) and RSM (*R* = 0.985320) in terms of accuracy, with an R-value of 0.997603 and the lowest MAPE of 0.0748. According to ANOVA results, peak current was the most significant WEDM parameter, accounting for 60.21% of the variation in surface roughness. The suggested method, combining support vector machine and ANN algorithm, can efficiently and accurately analyze and predict WEDM surface roughness on aluminum alloy C355 with Si₃N₄ and GNPs hybrid composites. Hence, this innovative study leveraged application of simulation, statistical and ML techniques to advance substrative manufacturing/WEDM process for the benefits of machining industries.

## Introduction

Aluminum hybrid nanocomposites are formed with different types of reinforcing particles. They are importance in the current trend of applications in the aerospace, automotive, marine, structural and energy storage devices, owing to their improved tensile strength, increased corrosion resistance, superior wear resistance, enhanced thermal steadiness and high impact strength^1,2^. The C355 alloy aluminum cast is known for its exceptional purity and ability to undergo heat treatment to age-harden. It has exceptional casting qualities, is suitable for both sand and permanent-mould casting techniques and is strongly advised to be used for pressure-tight castings. Cranks, propeller boxes and substantially pressured regions are only a few of the various components of automobiles and airplanes that use this alloy. The provided datasheet in a study goes into detail on its composition, physical properties, toughness, flexibility, yield strength, creep opposition, corrosion resistance and durability against fatigue^3^. The C355 aluminum alloy is commonly heat treated to reach its highest strength potential, because it shows exceptional strength retention at high temperature. This makes it especially suitable for operations, such as casting and continuous mould casting, in addition to applications that need pressure-tight qualities. When compared with the more popular casting metal A356, the C355 and 355 alloys are substantially stronger, because of the addition of copper^4^.

The physical, mechanical, tribological and microstructural characteristics of metal and polymer matrices are positively impacted by addition of some particles, such as C, Al_2_O_3_, TiC, Fe_2_O_3_, TiO_2_, SiO_2_, ZrC, SiC, MgO, B4C, CNTs, TiB_2_ and Gr according to a few studies^5,6^. This present investigation used two different types of nano reinforcements. They were silicon nitride nanoparticles (Si_3_N_4_NPs) and graphene nanoparticles (GNPs). High-performance ceramic silicon nitride (Si₃N₄) is renowned for its superior mechanical and thermal qualities. In some situations, the performance of metal and ceramic composites is greatly enhanced by the addition of Si₃N₄, as a reinforcement^7^. The specific benefits of Si_3_N_4_ reinforcements, such as high temperature stability, thermal shock resistance, low density, reduced weight, improved wear resistance, excellent thermal conductivity, high electrical insulation, biocompatibility and corrosion resistance are used in an assortment of productions, with aeronautical, defence, motorized, electronics, thermal, energy sector, tooling and cutting applications. They also retain strength and sharpness at elevated temperatures, are highly durable in abrasive and corrosive environments, and maintain structural integrity under high loads. The properties of composites are continuously improved by the incorporation of Si_3_N_4_, as a reinforcing material^8,9^. Composites have been made by incorporating Si3N4 particles into Al2219 aluminum alloy, using squeeze and stir casting. The results showed better yield strength, increased hardness, reduced ductility, increased compressive strength, increased impact strength, uniform distribution across the cross section and increased ultimate strength. The exceptional mechanical properties and strength of Al2219-Si_3_N_4_ composites make them perfect for application in the automotive and aerospace industries^7^. Shalby et al.^10^ and Arya et al.^11^ effectively developed a SiC/Si_3_N_4_ aluminum hybrid composite and reported that the combination of strengthening improved the composite materials tribological, mechanical and physical characteristics. The Cu–Sn/Si_3_N_4_ composite showed complete reinforcement interaction with the matrix, homogeneous distribution and no pores. In comparison with ordinary bronze alloy, the same sample exhibited low ductility value, high hardness, tensile strength and yield strength^12^.

Moving forward, graphene reinforcements in metal matrix composites (MMCs) are very important, because of their remarkable qualities, which improve the performance of the materials. In MMCs, graphene specifically offers the following advantages: increased mechanical strength, enhanced resistance to wear, improved thermal conductivity, lightweight characteristics, resistance to corrosion, electrical conductivity, adaptability and nano structuring potential^1,2,13^. Aerospace, automotive, electronics and electrical components, energy storage, biomedical, military, maritime, sports and recreation are just a few of the industries that employ graphene in metal composites, owing to its remarkable physical, thermal, electrical and chemical properties^14,15^. GNPs were added to Al6061 alloy in varying weight percentages (wt%), and a stir casting procedure was employed to manufacture the composite. Improved mechanical qualities, including higher yield and eventual ductile forte and rigidity, in totalling to microstructural measurements showing random distribution of nanocomposites, were the advantages of the Al6061/GNPs composites^1^. The composites of graphene nanoplates and aluminum alloy were formed using two distinct manufacturing techniques, including stir casting and ball milling. Reduced grain size and increased ultimate tensile strength were obtained by adding graphene nanoplates to composites. The preferred route for crack formation was determined to be the largest concentration of graphene nanoplates, agglomerating on grain boundaries^16^. When graphene is added to aluminum alloy composites using powder metallurgy, the results demonstrate that the graphene is dispersed at the boundary and forms a strong bond with the aluminum in the Gr/Al composite. The mechanical properties of the composite also improve, as does its resistance to corrosion and wear^17^.

When related to the base alloy matrix, the tensile strength of the GNP-reinforced aluminum produced by ultrasonic vibration stir casting method was improved. The activity of GNP in the aluminum matrix, which fortifies and refines the grain, is responsible for their improved mechanical qualities. Wettability in poor, non-uniform particle dispersion and the creation of weak intermetallic compounds were identified as some disadvantages in comparable investigations conducted in stir casting^10,12,15,16^. Therefore, this current study has decided to employ dual step stirring, preheating and the addition of C355 aluminum alloy to attain the desired results.

Besides, wire electrical discharge machining/machined (WEDM) is an unorthodox machining technique that cuts material by repeatedly producing electrical impulses. In contrast with traditional processing, no typical tool is utilized. The machine uses a wire electrode of 0.4 mm diameter, which is often composed of metal matrix composite, copper or brass^18^. As a result, soft and thin-walled items can be machined, as the machining process does not involve applying mechanical forces to the workpiece. The sole requirement for machinability is that the workpiece have a minimal electrical conductivity, because the machining is done using electrical impulses. This enables the machining of any materials, irrespective of its physical or mechanical characteristics. Particularly for the machining of materials that are often challenging to mill, this is a highly prized attribute. The automotive, energy, food, medical and military sectors are just a few industries that employ WEDM extensively^19^. The maximum strain toughening feature leads to poor contraption using traditional approaches, which causes problems such as burr formation, rapid tool wear, low surface quality and the formation of ribbon-like and tangled chips. WEDM is an innovative machining process that has been offered as a substitute for regular machining to overcome the afore stated restrictions^20,21^. Wire electrical discharge machining was only used in Prabhakar et al. to precisely cut AA8090 plates for specimen preparation; it was not used for surface integrity assessment or machining analysis^22^.

In process simulation and optimization, computational methods including artificial neural networks (ANNs), support vector machine (SVM) and genetic algorithm (GA) have gained a lot of traction, because of their excellent prediction capabilities and outstanding level of precision. These strategies are very dependable for increasing operational effectiveness and potential to produce favourable outcomes faster than statistical approaches^23^. Numerous domains have employed machine learning (ML) algorithms to forecast the results of costly and time consuming trials that are challenging to ascertain analytically. Civil engineering^24^, biology^25^, energy^26^ electrical engineering^27^, health^28^, materials technology^29^, industrial and production engineering^30^ and physics^31^ are a few examples of these fields. Furthermore, extreme learning machines (ELM) and support vector regression (SVR) were both successfully used in these domains^32^. In the current investigation, the WEDM variables were modeled, using both SVM and ANN approaches. GA was used to obtain the best results for adjusting the process variables to consider the accuracy of the WEDM holes on aluminum 6061^33^. The formulation of intelligence manufacturing principles, which can thus be used in the prediction and augmentation of WEDM, and other machining techniques are obtained on the implementation of ML structures and strategies^34^. The ML models supported response monitoring of WEDM processes easier by considering the various end-user expectations and addressing the external elements that affect the process^35^. The WEDM of super alloy of 605 has been experimentally studied, and it is advised to employ sophisticated ML algorithms and optimization techniques, including SVM and GRA^36^. For the required surface polish and plates dimension, the cutting speed, spark gap and current were predicted, using two different ML approaches: ANN and SVM^37^. ANN was employed to estimate surface roughness and acoustic emission (AE) data in titanium WEDM after observing that SVM generates improved prediction than ANN^38^. The efficacy of the model was checked by utilizing the root mean squared error and coefficient of correlation. The results demonstrate that the SVR approach and scikit-learn-based linear regression outperform the other methods in terms of accuracy in forecasting^39^. In another research on biodegradable titanium alloys, Sharma et al. used the SVM, Gaussian process regression (GPR) and a ML-based method to simulate the surface roughness and discovered that it was helpful for simulating reactions for intricate processes^40^. Random forest and SVM approaches were applied by Raj et al. to simulate reaction when the titanium alloy was processed, using WEDM^41^. With SVM approach, Singh et al. developed a prediction model for aerospace and structure grade alloys that undergo wire EDM processing^42^. In another study, the surface roughness (*R*_*a*_) of WEDM Mg-SiC nanocomposite was predicted and optimized, using the SVR with GA and particle swarm optimization (PSO) models. The outcomes indicated that pulse-on time was the primary determinant of *R*_*a*_ and that *R*_*a*_ improved with pulse-on time. A considerable boost in accurate forecasting was obtained when genetic algorithms were integrated into the employed SVR for generating a sophisticated model for forecasting^43^. For the purpose of to increase energy efficiency and productivity, this work combines deep learning and metaheuristic optimization to injection moulding. This allows for intelligent material prediction and multi-objective cooling parameter optimization^44^.

This study uses Taguchi-based gray relational analysis and ANOVA to achieve multi-objective optimization of friction stir welding parameters and enhance the tensile strength and impact energy of Al-2024 joints^45^. Tamang and Chandrasekaran worked was aimed at developing and validating response surface models for the optimization of machining parameters. It shows how quadratic regression and design of experiments can reveal parameter interactions that significantly affect surface finish and productivity^46^. This study integrates hybrid Grey-ANFIS machine intelligence in optimizing EDM machining of TiN-Si₃N₄ composites by improving predictive accuracy, demonstrating advanced modeling in non-conventional processes^47^.

The published literature makes it evident that while aluminum-based metal matrix systems and hybrid nanocomposites have been thoroughly studied, there are still few studies that address surface integrity caused by machining. There is little research on wire electrical discharge machining (WEDM) of aluminum hybrid nanocomposites, and what is available is mostly limited to monolithic alloys or composites reinforced with a single secondary phase. Furthermore, the majority of current studies rely on traditional statistical methods, which are frequently insufficient to capture the nonlinear interactions between surface roughness and WEDM process parameters. In a number of cases, WEDM has been used more for specimen preparation than for the methodical assessment of surface features caused by machining. As a result, there are still few solid and trustworthy predictive models available for evaluating the WEDM surface roughness of silicon nitride–graphene hybrid nanocomposites based on C355.

In order to fill up the gaps in knowledge on the subject, this work systematically carries out the surface roughness characteristics associated with the WEDM of stir-cast C355/silicon nitride/graphene hybrid metal matrix composites. The composites are developed using a controlled stir-casting method with varying reinforcement proportions. A combined experimental and computational approach is selected, employing the effect of important WEDM variables—pulse on-time, pulse off-time, peak current, servo voltage, and feed rate—based on the prime importance associated with the discharge energy and material removal characteristics. Surface roughness is identified as the major output response for the analysis of the surface quality associated with machining. Predictive modelling has also been done using comparative models based on artificial neural networks & support vector regression approaches with RSM for bench-marking. The machined surface characteristics are also analysed using field-emission scanning electron microscopy. The significance of this work is the combined analysis of the surface integrity associated with the machining characteristics of the WEDM and the comparative predictive intelligent modelling associated with the developed silicon nitride and graphene hybrid nanocomposites based on C355.

## Experimental procedures

### Selection of matrix and reinforcements

C355 aluminum alloy was used in this study. It is commonly heat treated to reach its highest strength potential, because it shows exceptional strength retention at extreme temperatures. This qualifies it to be specifically appropriate for applications, such as casting and usages that call for pressure tight qualities. The addition of copper to the C355 and 355 aluminum casting alloys greatly increases its strength in comparison with the more widely used aluminum casting alloys. The C355 alloy can provide a strong, durable casting that is appropriate for challenging uses, including parts of engines and compressor cases. The chemical compositions of C355 aluminum alloy used are presented in Table 1.

**Table 1:** Chemical compositions (wt%) of the C355 aluminum alloy in used in the investigation.

Elements	Si	Cu	Mg	Mn	Fe	Zn	Al
wt%	6	1.6	0.8	0.2	0.3	0.2	Others

Si_3_N_4_NPs significantly contribute to the properties of the aluminum alloy composites by improving their mechanical, thermal and chemical characteristics. The modifications that contributed to their values attract extensive application by several industries, considering their crucial good strength, better wear resistance and durability. Another strengthener is GNPs. It was added to the composite samples at 0, 6 and 12 wt%. GNPs improve the tensile strength, yield strength and stiffness of aluminum alloys by acting as effective reinforcement. Their high intrinsic strength, high wear resistance and modulus contribute to the overall performance of the composites. Load bearing capability is especially advantageous for many situations in the aerospace, automotive and marine industries. The accumulation of GNPs can improve the electrical conductivity of aluminum composites, making them useful in electronic components and conductive materials. The two-dimensional (2D) structure of graphene helps to arrest crack propagation, improving the fracture toughness of the composite. The characteristics of the strengthening elements are shown in Table 2.

**Table 2:** Characteristics of the strengthening elements.

Reinforcements	Size (µm)	Density (g/cm^3^)	Decomposition temperature (^o^C)	Hardness (HB)	Thermal conductivity (W/mk)
Si_3_N_4_NPs	36	3.14	1850	22	14.4
GNPs	40	2.24	4650	104	625

### Preparation of C355/silicon nitride/graphene hybrid metal matrix composites

**Fig. 1:**
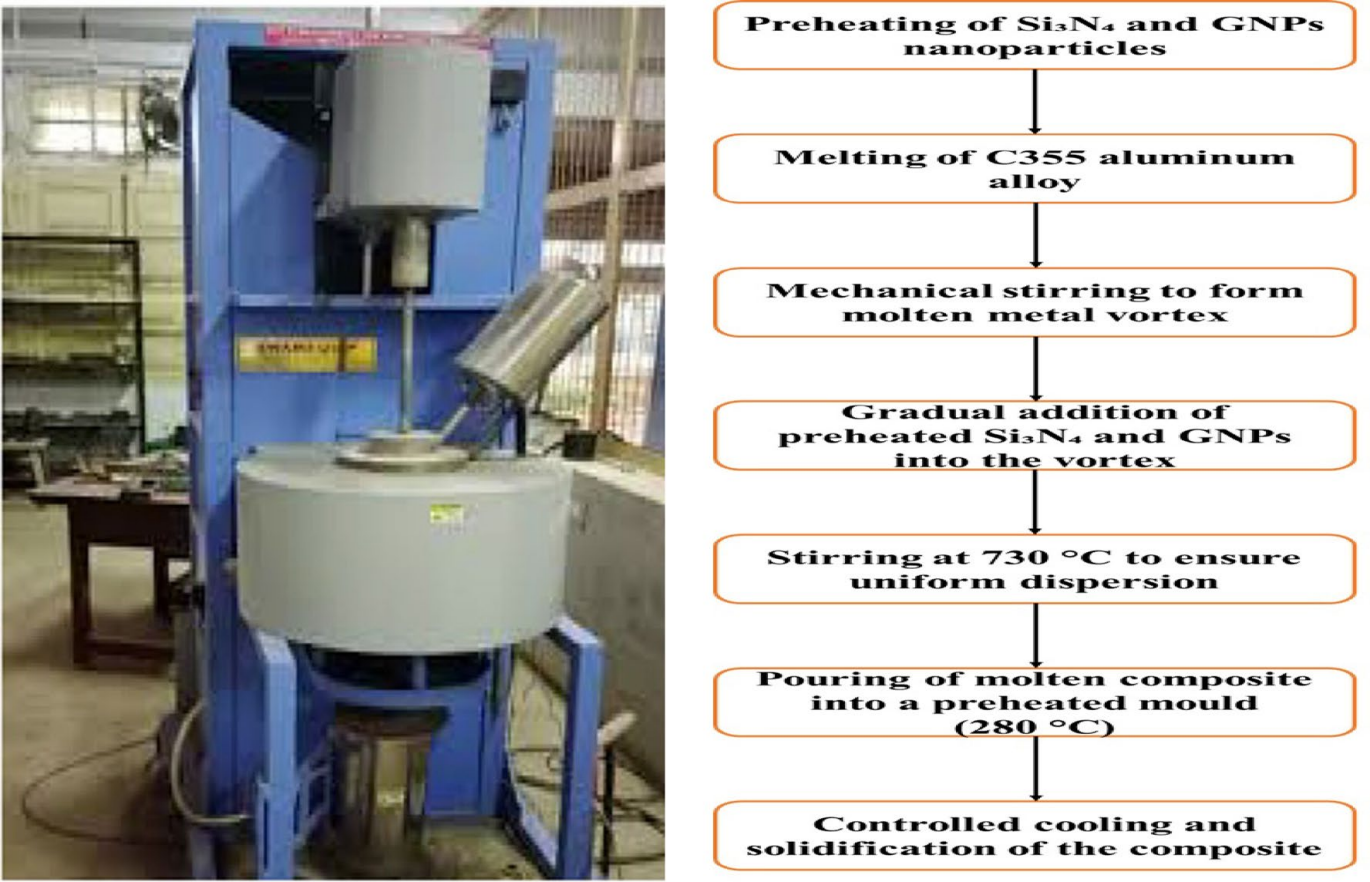
(**a**) Composite preparation set-up, using stir casting technique and (**b**) Experimental process of C355/silicon nitride/graphene hybrid metal matrix composites.

Stir casting method was used to manufacture the nanocomposites. Figure 1 shows the stir casting set up for production of varied proportion of 0, 6 and 12 wt% of GNPs in C355 aluminum alloy/Si_3_N_4_NPs/GNPs hybrid metal matrix composites with base matrix C355 aluminum alloy. The base metal, C355 aluminum alloy, was used as matrix. The reinforcement materials, Si₃N₄NPs and GNPs, were prepared by preheating to remove moisture and improve their wettability with the molten metal. The base metal was placed in a ceramic receptacle and frenzied in a resistance or induction incinerator to a temperature of 760 °C until it was fully a molten material. To remove dissolved gases that could cause porosity, a degassing process was performed, using argon or nitrogen gas. A mechanical stirrer with a graphite-coated blade was inserted to the liquefied metal to create a vortex. The stirring progression was maintained at a speed of 450 RPM for about 10 min to ensure uniform mixing. The preheated Si₃N₄NPs and GNPs were then gradually added to the vortex to ensure even dispersion. The stirring temperature was maintained at 730 °C to prevent premature solidification. Once the reinforcement particles were evenly distributed, the molten composite was poured into a preheated mould of 280 °C to reduce thermal shock. Controlled cooling was allowed to ensure proper solidification and minimize defects, such as porosity and shrinkage.

### WEDM of C355/silicon nitride/graphene hybrid metal matrix composites

**Fig. 2:**
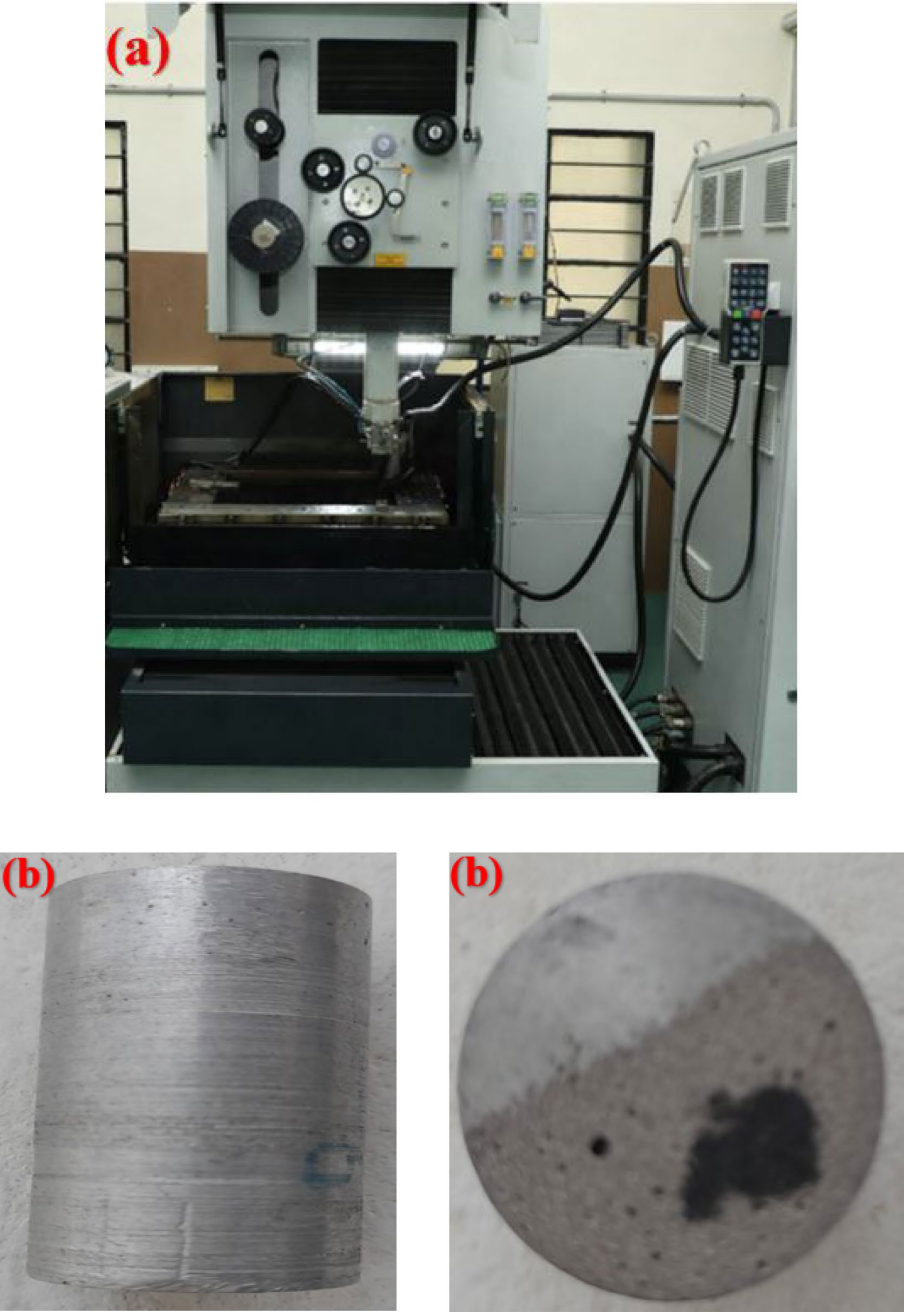
(**a**) WEDM machine setup and (**b**) WEDM workpiece samples.

A non-contact heat machining technique, called WEDM, was used to precisely cut electrically conductive material samples. It works by generating controlled electrical discharges (sparks) between a workpiece (C355/silicon nitride/graphene hybrid metal matrix composite) and the thin wire electrode, removing material through melting and evaporation. Figure 2 depicts the WEDM machine setup and workpiece samples.

The workpiece material was selected and prepared, ensuring it has electrical conductivity to enable spark erosion. The workpiece was cleaned to remove dust, grease or oxidation layers that might affect machining. It was clamped firmly on the WEDM machine table to avoid vibration or misalignment during cutting. Deionized water (dielectric fluid) was circulated in the machine to cool the workpiece and flush away debris. A thin wire electrode was selected based on the workpiece material and required precision. The wire diameter was 0.25 mm, depending on the cutting accuracy needed. Important machining parameters were set in the control system, including pulse-on time (Ton) and pulse-off time (Toff), peak current (Ip), servo voltage and feed rate. The WEDM testing environment variables and their levels are presented in Table 3, while their corresponding surface roughness measurement results are later provided in Table 4, under sub-Sect. 4.1 on results and discussion.

**Table 3:** WEDM process parameters and testing conditions.

Variables/levels	Pulse-off time (µs)	Pulse-on time (µs)	Servo voltage (V)	Feed rate (m/min)	Peak current (A)
1	60	120	35	2	14
2	65	125	45	4	18
3	70	130	55	6	22

The wire electrode was continuously fed from a spool and guided by pulleys to move along a programmed tool path. The wire did not touch the workpiece; instead, electrical discharges (sparks) occurred in the small gap between them, generating intense localized heat. The heat melted and vaporized the substance, which was then removed by the dielectric fluid. The system precisely controlled the movement of the wire to achieve complex shapes, contours and fine features. Once the required profile was cut, the machine stopped and the nanocomposite sample (workpiece) was carefully removed. The machined part was cleaned to remove debris and dielectric fluid residues. A surface inspection was performed using measuring techniques, including SEM to check for accuracy and surface quality.

## Simulation techniques

### SVR simulation

**Fig. 3:**
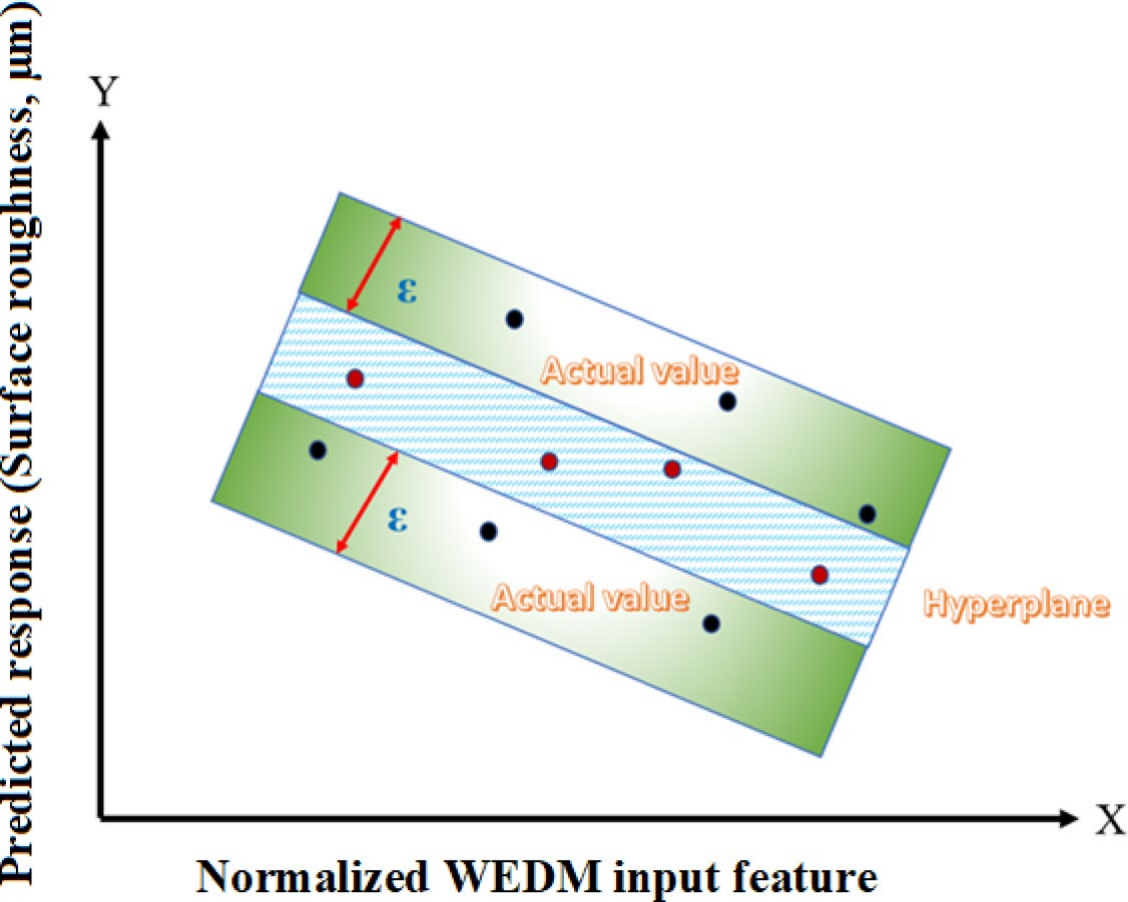
The regression hyperplane and ε-insensitive area are displayed in the SVR simulation.

The SVR computation simulation of surface roughness was made possible by the execution of the Libsvm option used in MATLAB software interface. Although SVR algorithms very greatly to hyperplane variables, including gamma, box constraint, epsilon, and kernel function, determining the most appropriate hyperplane variables before simulated proved essential. Figure 3 displays the nonlinear SVR simulated in an enlarged perspective. The figure successfully illustrates the main objective of the SVR simulation, namely, to acquire the highest number of locations inside the epsilon () range by utilizing the most suitable or optimal hyperplane variables. The optimal values of a number of hyperplane variables, including C, epsilon, degree, gamma, and kernel function, comprised 10, 0.01, 3, 0.2, with “linear” correspondingly. Training and testing data were separated into 60 and 40% of the data used for experiments in grid search, accordingly. A grid search approach has been adopted for optimizing hyperparameters of SVR algorithms such as the penalty term (C), gamma value (γ), epsilon (ε), and power value. For each value selected for these hyperparameters, an SVR algorithm is used for model and results evaluation based on MSE and R value criteria. The best hyperparameters obtained are those having least MSE and highest R value. Prediction errors were noted when data was trained, using various hyperplane settings during grid search. The model that had the lowest mean squared error (MSE) during testing and training was selected, indicating that its predictions were closer to the actual values and suggesting better performance. Within the end, surface roughness had been calculated with the optimal SVR simulated settings.

### ANNs simulation

The detected surface roughness in WEDM C355/Si_3_N_4_NPs/GNPs composites was estimated, using an array of layers feed forward ANN. For this, the MATLAB R2024b neural network software toolbox was used. The data was separated within training, testing and validation proportions of 5:1:1 corresponding to 71.4%, 14.3%, and 14.3%, respectively. The network was trained, using the Levenberg-Marquardt (trainlm) backpropagation technique. All the layers were thought to be activated by the hyperbolic tangent sigmoid function (tansig), which created nonlinear relationships between the independent and dependent parameters. Surface roughness was associated with the WEDM process, which used the following inputs: pulse-on time (µs_on_), pulse-off time (µs_off_), peak current (A), servo voltage (V) and wire feed rate (m/min). The statistical metrics of MSE and average error (AE) between target and prodicted values were used to construct the best neural network architecture. Equations (1) and (2) were used to calculate the AE and MSE, respectively.

1 $$\:AE=1/n\sum\:_{i=1}^{n}(Si-Spi)$$

2 $$\:MSE=1/n\sum\:_{i=1}^{n}(Si-Spi)^{2}$$

where $\:Si$ represents experimental data,$\:\:Spi$ denotes ANN prophesied dataset and $\:n$ stands for the total data sets.

### RSM simulation

RSM, a form of statistical analysis, was implemented to establish a relationship between the initial and final limits in a scientific calculation. The RSM simulation was fitted with the WEDM process parameters and corresponding surface roughness. Additionally, analysis of variance (ANOVA) was utilized to determine how input factors affected surface roughness. A key parameter was identified by calculating the percentage contributions of the various components to the variance in surface roughness. The correlation coefficient (R-value) between the anticipated and experimental outcomes was employed to assess the developed regression simulations effectiveness.

## Results and discussion

### Surface topography

The WEDM experimental measured surface roughness values obtained are presented in Table 4. The WEDM experiment was planned using a Taguchi orthogonal array L27 (3⁵). The table comprises five control factors and three levels for the factors, which gives a total of 27 experimental runs. Therefore, the L27 (3⁵) design was chosen to study the individual and interaction effects of five key WEDM parameters over three practical operating levels in a very effective way, with a considerable reduction in experimental cost and time compared to a full factorial design. These numerous data can be better understood when they are concomitantly considered with FESEM images, as subsequently discussed.

**Table 4:** Experimentally measured surface roughness values obtained from WEDM experiments.

WEDM exp. no.	Pulse-off time (µs)	Pulse-on time (µs)	Servo voltage (V)	Feed rate (m/min)	Peak current (A)	Surface roughness (µm)
1	60	120	35	2	14	0.746
2	60	120	35	4	14	0.782
3	60	120	35	6	14	0.805
4	65	125	45	2	14	0.823
5	65	125	45	4	14	0.864
6	65	125	45	6	14	0.891
7	70	130	55	2	14	0.926
8	70	130	55	4	14	0.947
9	70	130	55	6	14	0.983
10	70	125	35	2	18	1.023
11	70	125	35	4	18	1.064
12	70	125	35	6	18	1.087
13	60	130	45	2	18	1.123
14	60	130	45	4	18	1.147
15	60	130	45	6	18	1.169
16	65	120	55	2	18	1.184
17	65	120	55	4	18	1.209
18	65	120	55	6	18	1.246
19	65	130	35	2	22	1.267
20	65	130	35	4	22	1.283
21	65	130	35	6	22	1.306
22	70	120	45	2	22	1.348
23	70	120	45	4	22	1.372
24	70	120	45	6	22	1.394
25	60	125	55	2	22	1.416
26	60	125	55	4	22	1.434
27	60	125	55	6	22	1.458

**Fig. 4:**
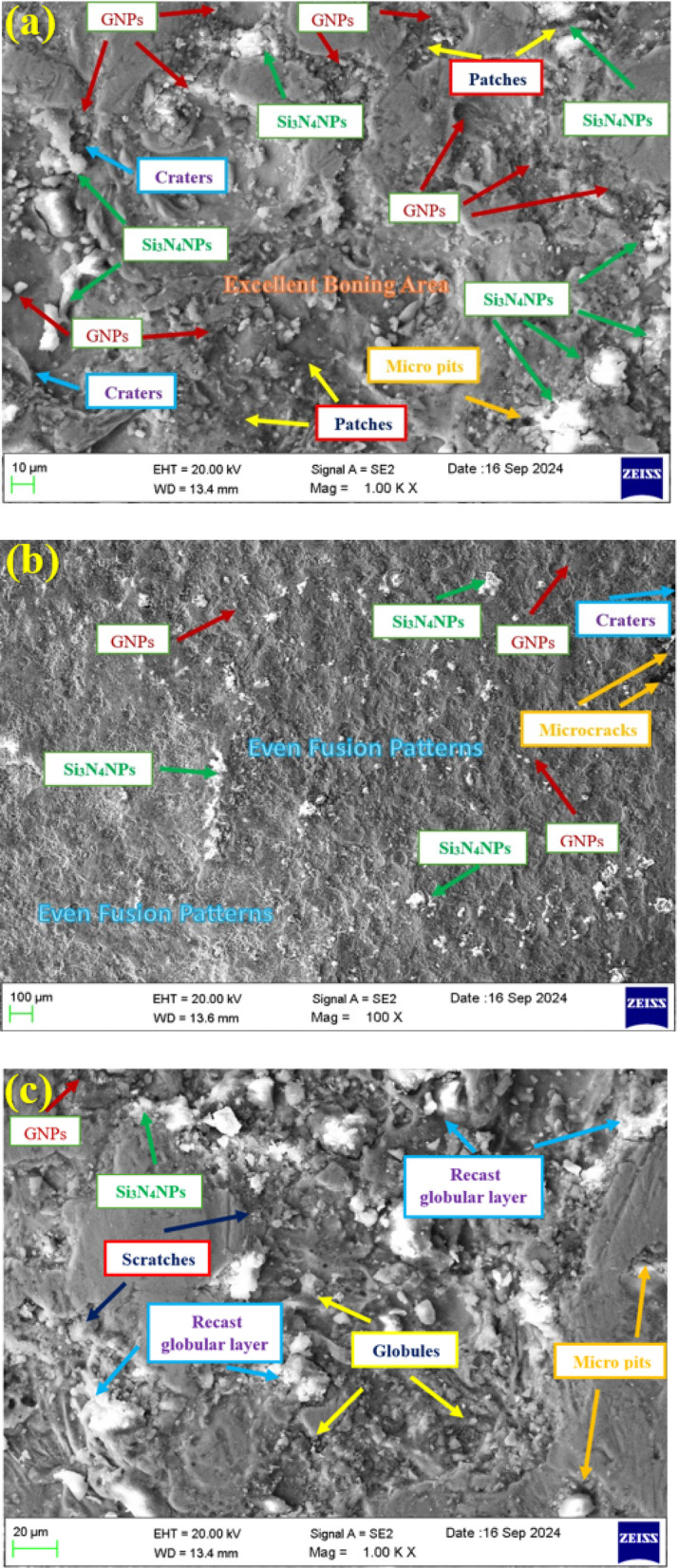
WEDM surfaces of (**a**) 0, (**b**) 6 and (**c**) 12 wt% C355/silicon nitride/graphene hybrid nanocomposite samples.

Therefore, Fig. 4 shows field emission scanning electron microscopy (FESEM) images of WEDM machine samples of C355/silicon nitride/graphene hybrid nanocomposites. The silicon nitride and graphene nanoparticles were clearly mixed in the composite; therefore, resulting in an excellent bonding of matrix and nano reinforcements. The effect of the presence of graphene on the process of electrical discharge in the WEDM process has been expected in the literature. However, the measurement of electrical conductivity is not carried out within the current study; it can therefore be noted as qualitative, as it is inferred from the machining performance and other research works. The FESEM further depicts that silicon nitride and graphene nanoparticles dispersed more rapidly when compared with C355. Additionally, discharge current increased with electrode wear, due to the higher transfer of electrical energy into the machining gap. FESEM was used to examine the surface roughness of the C355/silicon nitride/graphene hybrid nanocomposites machined with copper, copper and nanocomposite electrodes or tools, which were 0.864, 1.123, and 1.458 μm, respectively. Figure 4(a) shows the surface topography of C355/silicon nitride/graphene hybrid nanocomposites cut with copper tool, having dark patches, craters and micro pits. Additionally, the surface showed a re-melted layer that resulted from insufficient flushing. Figure 4(b) depicts surface topography with microcracks, craters and even fusion patterns at higher magnification. It can be deduced that using copper as an electrode did not entirely disperse the heat. Pock marks that were created by the discharge of trapped gas during the cooling phase were observed on the surface. Observation of the presence of recast globular layer on the WEDM surface achieved with the C355/silicon nitride/graphene hybrid nanocomposite tool electrode was a noteworthy characteristic, as depicted in Fig. 4(c). The recast globular layer was white, round and dispersed throughout the whole surface. In addition, the machined surface contained scratches and globules. The recast globular layer appeared had a lot of little melted layer at greater magnification, as observed in Fig. 4(b). Due to this, the A1 composite tool provided a 61% poorer surface than the copper electrode. Additionally, globules and micropits were observed on the surface.

### SVR Simulation

A grid search was used to find the optimal hyperplane settings. For grid lookup, the conditions previously presented in Table 5 were utilized as hyperplane variables. From the results acquired through grid search, it was seen that a linear kernel was the best choice for training an SVR classifier, as it resulted in the least prediction error and yielded the highest value of the correlation coefficient among other kernels, including non-linear ones.

**Table 5:** SVR input parameters determined using the grid search method.

hyperplane variables	Optimal values
C	100
epsilon	0.00100
gamma	0.4
Degree	4
Kernel	Linear

**Fig. 5:**
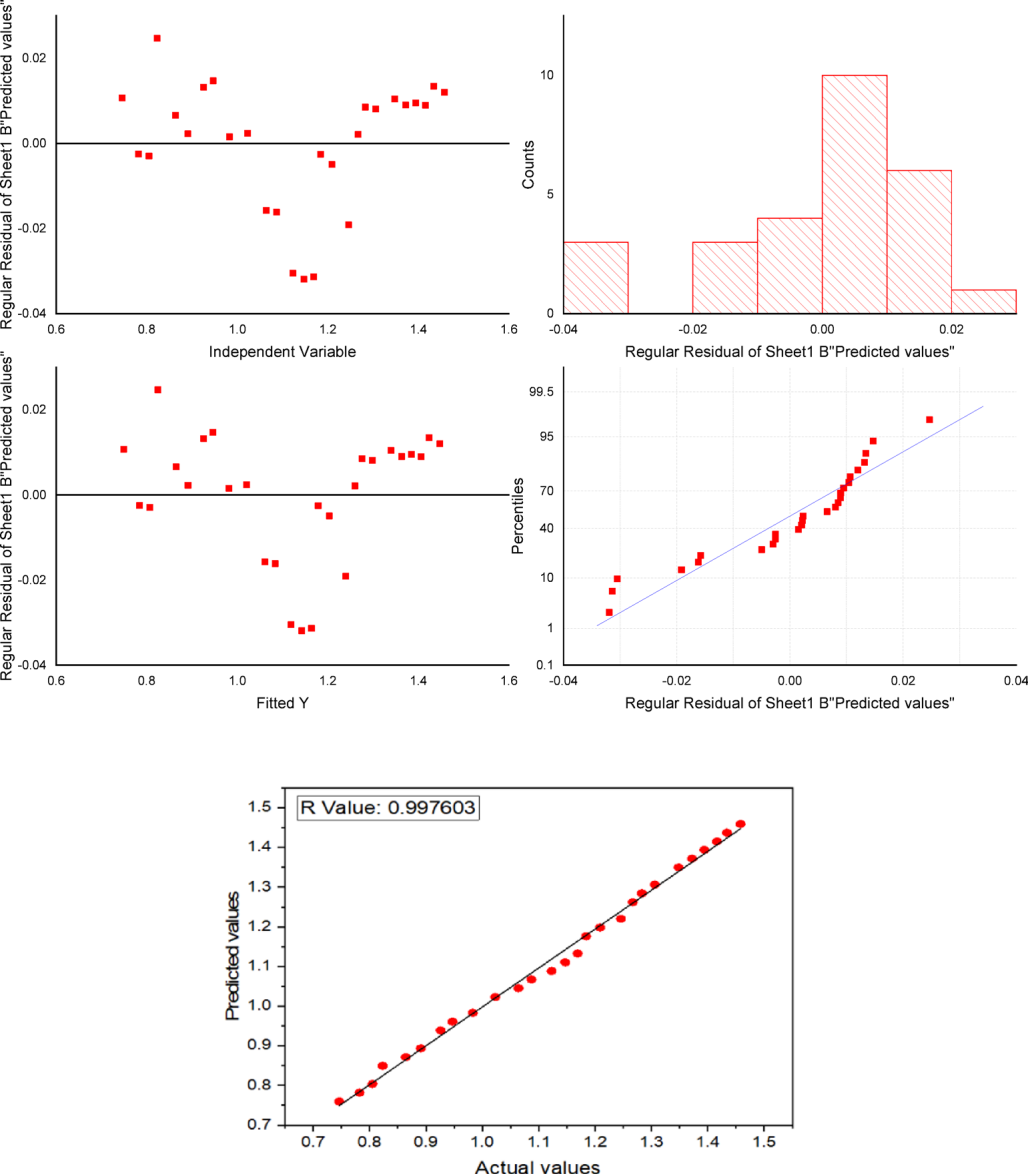
Correlation between experimental and SVR simulation predictions.

SVR Results in this study only relate to SVR on the linear kernel-based model shown in Table 5. In order to identify the ideal settings that produced a small MSE and an elevated R-value, a simulator was developed using 55% of the datasets. For the purpose of obtaining an appropriate fit for the computer model, the hyperplane settings were changed. The libsvm software layout was leveraged for implementing the SVR simulated within ideal settings. Towards expedite the process and avoid zero items, the collected information was initially turned into minimal information. When the optimal support level vectors were accomplished, the SVR simulator received training. At the 26th cycle, the algorithm had 18 support vectors, demonstrating its remarkable accuracy. Consequently, the training ended with the 26th cycle, and the learnt simulated was used to estimate the surface roughness. Concurrently, the experiment’s quality was premeditated utilizing the R-value and MSE. With an overall R-value of 0.997603 and an MSE for training and testing data of 2.875624e^− 04^ and 1.248754e^− 04^, correspondingly, the SVR simulated displayed outstanding accuracy in forecasting. Figure 5 shows an independent variable, fitted, regular residual predicted and correlation graph, plotting real values against SVR projected values.

### ANNs simulation

**Fig. 6:**
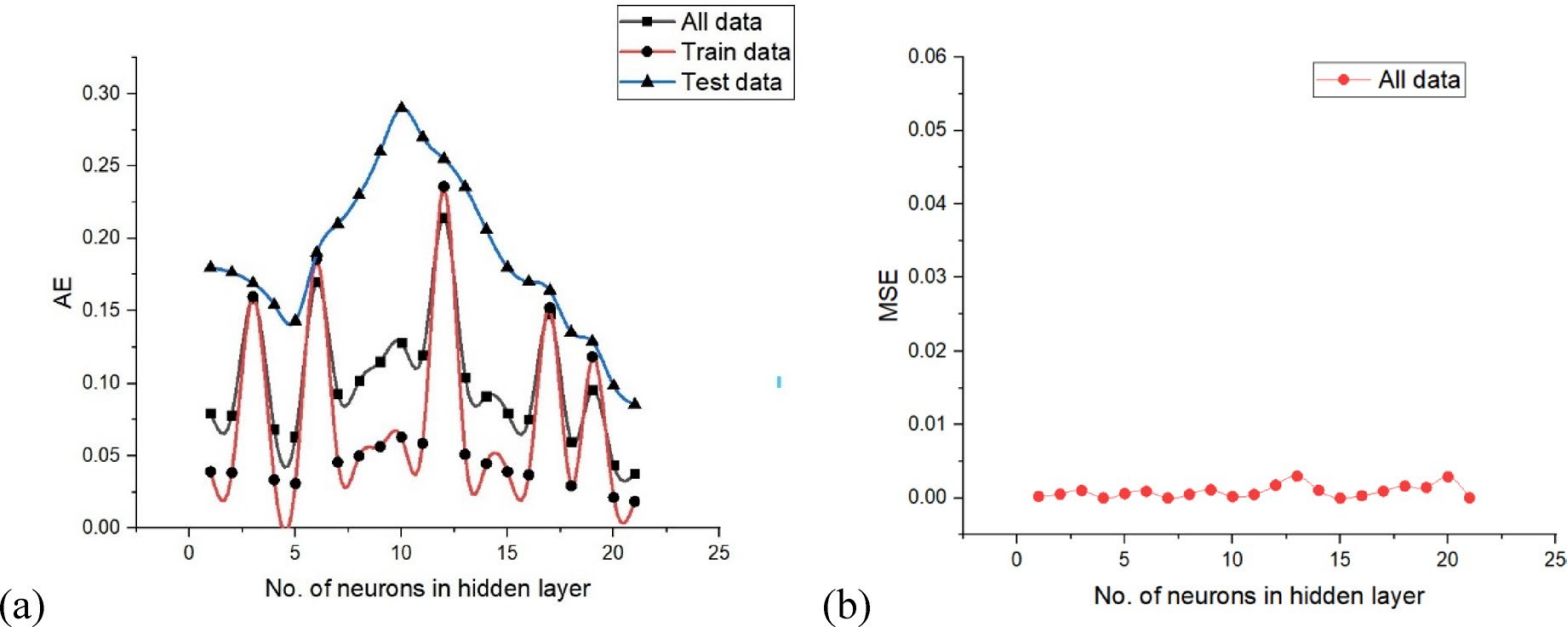
(**a**) AE and (**b**) MSE in a single hidden layer *versus* different neurons.

**Fig. 7:**
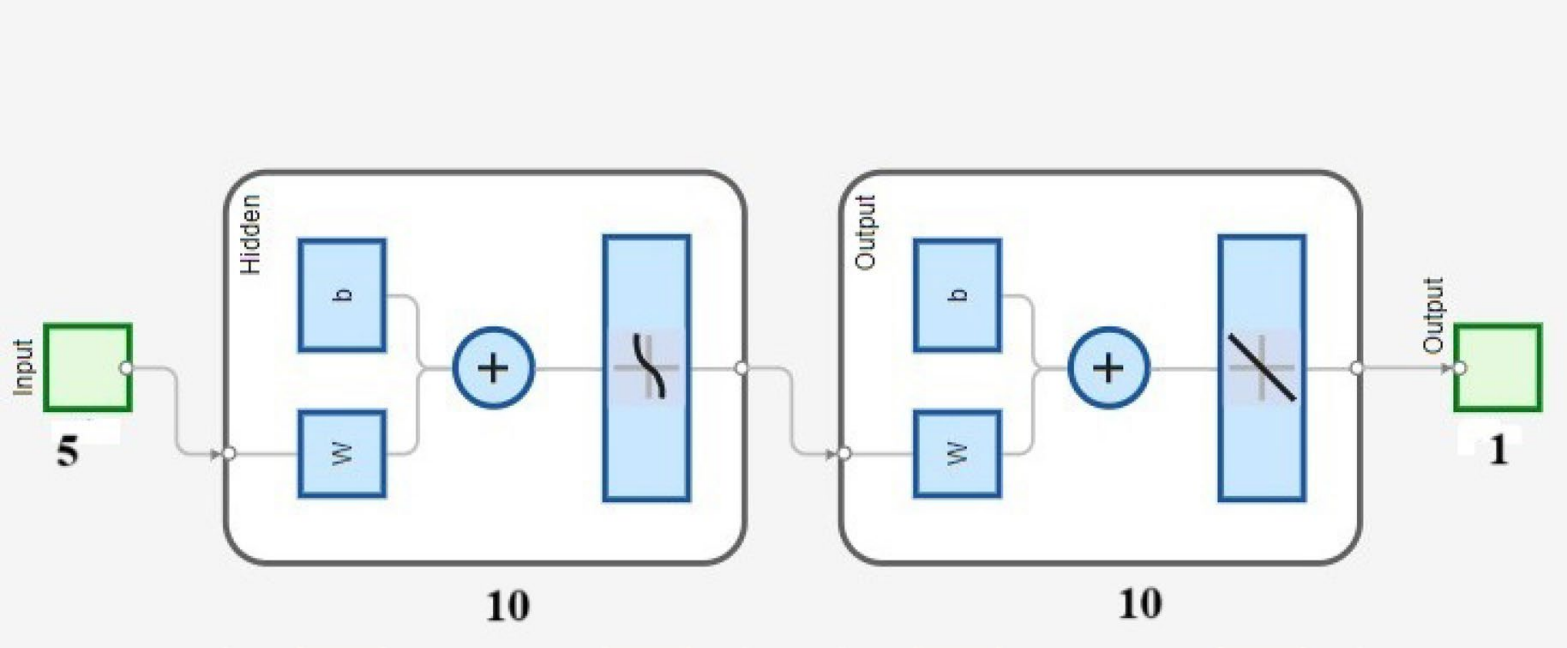
Schematic representation of the developed optimal ANN design.

The optimal neural network design was identified by evaluating the AE and MSE across the test, train and overall datasets. The simulation underwent training, testing and validation with different neuron and layer configurations, and the error for every neuron was computed accordingly. Figure 6 exhibits the AE and MSE for various neuron counts in only hidden layers. ANN with dual hidden layers, each containing 10 neurons, achieved the lowest AE and MSE values of 0.2968 and 0.0300, respectively, for the test data. Comparing various neural network topologies, it was also observed that AE and MSE were significantly low for both train and all data. To forecast the surface roughness, a neural network simulation with a topology of 5 10-10-1 was used. Figure 7 shows a schematic representation of the developed optimal ANN design. Later, a few iterations were used to train the produced ANN simulation once again to prevent overtraining. AE and MSE were computed at different iteration count and their variations with iteration count to monitor the effectiveness of the model. The AE and MSE test, train and overall data were observed to be at their lowest at 14,000 iterations, relatively constant until 16,000 iterations and then started to increase significantly at 19,000 iterations. Lastly, by comparing expected and actual results, the R-value was used to assess performance of the ANN simulation, and the association graph for the created ANN simulation.

**Fig. 8:**
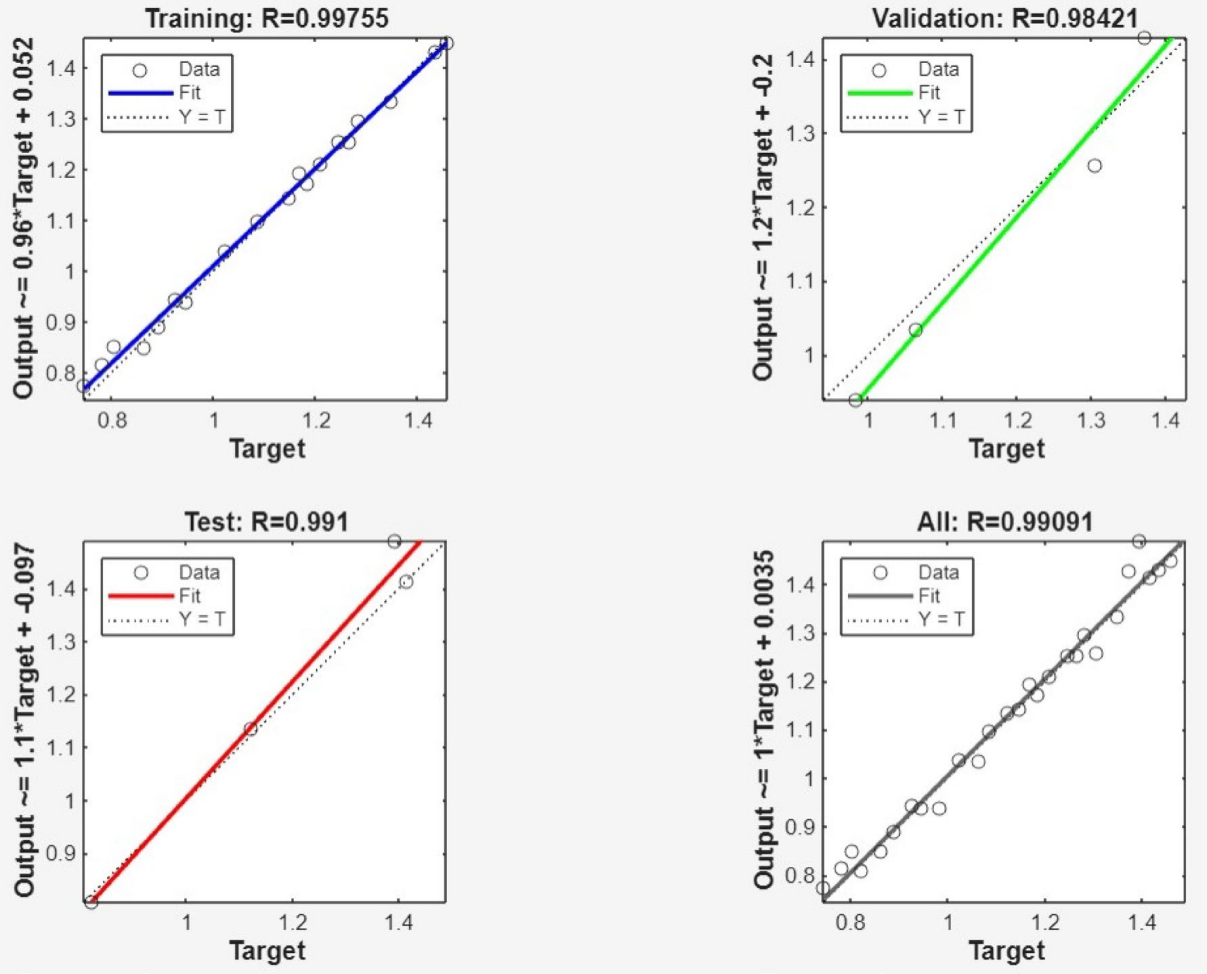
Association between experimental results and ANN simulation estimations.

**Fig. 9:**
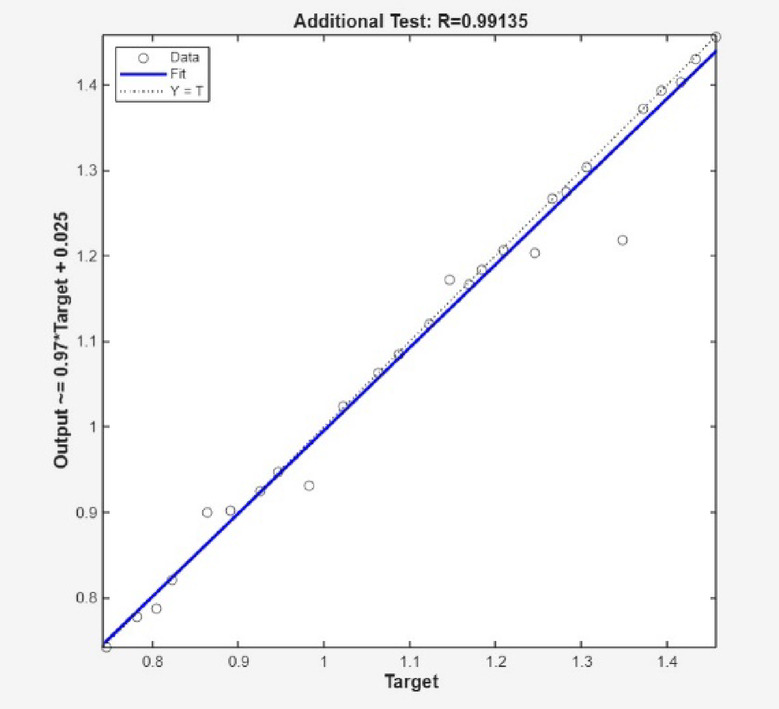
Additional test.

**Fig. 10:**
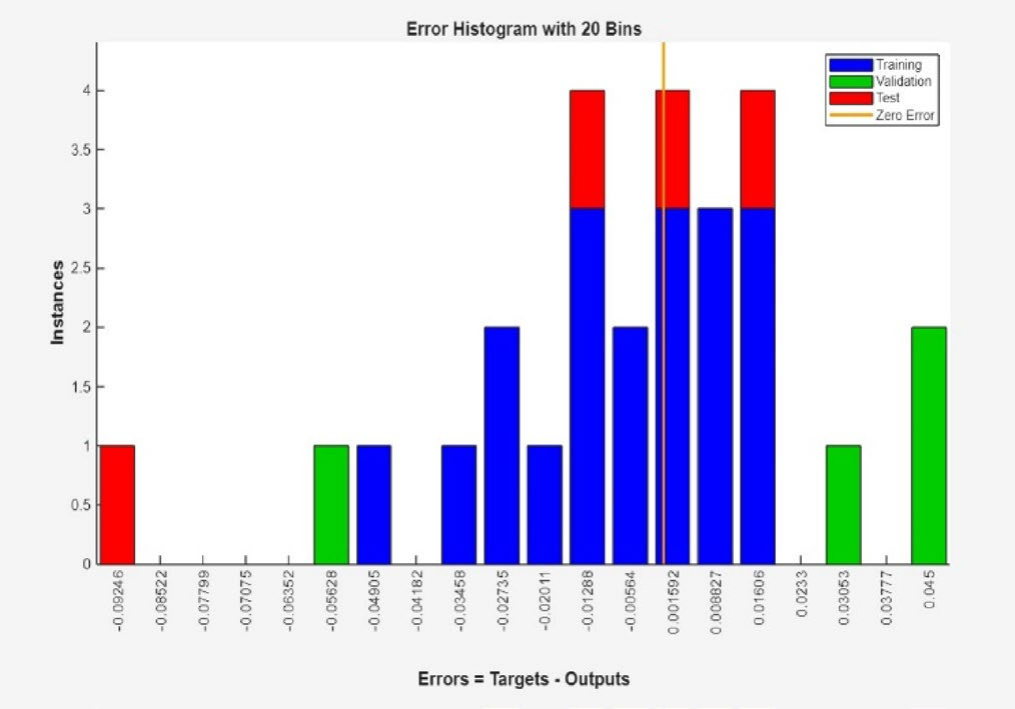
Comparable R and error values.

**Fig. 11:**
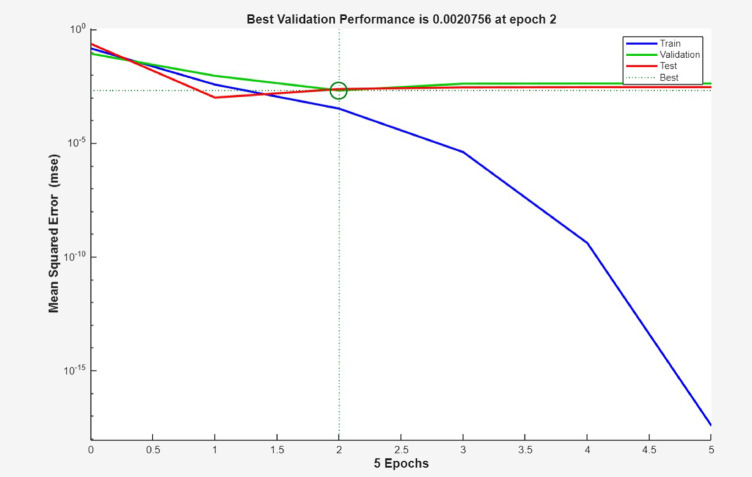
Optimal validation.

More also, Fig. 8 clearly shows the high accuracy of the ANN simulation, as evidenced by the R-values of 0.99980, 0.99932 and 1.00000 for training, test and validation data, respectively. The R-value for all data was 0.99988, signifying a strong association between the ANN predicted and the experimental surface roughness values. The addition test is shown Figs. 9 and 10, which were likewise matched with comparable R and error values. Figure 11 depicts that MSE decreased with the number of epochs for optimal validation. The finest recital was obtained for surface roughness (SR), at an MSE values of 2.0e^− 06^, indicating high prediction accuracy.

Even though the achieved prediction accuracy was high, the size of the dataset was limited to 27 experimental runs. In order to avoid overfitting, the ANN model was trained by using separate training, validation and testing datasets. The model convergence was monitored through the trend in validation error. Error increases beyond 16,000 iterations (Fig. 11) were used as the stopping criterion in order to avoid overtraining. The architecture selected ANN was 5–10-10-1 based on minimum validation error rather than based on solo training accuracy.

### RSM simulation

Based on experimental data, the RSM simulation for surface roughness obtained in WEDM C355/silicon nitride/graphene nanocomposites was developed. A second-order polynomial equation, including independent parameters and their interactions, was developed. Equation (3) presents the created RSM equation that established the connection between surface roughness and several contribution parameters, including servo voltage, pulse-on time, peak current, wire feed rate and pulse-off time. With the help of known coefficients, the constructed RSM model shows an exact mathematical relationship that can be utilized to estimate surface roughness.

3 $$\begin{aligned}&SR =380 - 5.26 P on time - 2.49 P \:off \:time + 0.33 P \:current + 0.73 Servo \:voltage \\&+ 3.96 Feed\: rate + 0.0154 P\: on\: time*P on time + 0.00218 P off time*P \:off \:time \\&- 0.0406 P \:current*P \:current - 0.00022 Servo voltage*Servo \:voltage \\&- 0.0665 Feed\: rate*Feed \:rate \end{aligned}$$

The large constant term in Eq. (3) is due to the use of coded variables in the regression analysis and is not a measure of any real surface roughness value. The purpose of Eq. (3) is purely for predictive modeling on the coded space. Based on the experimental design, a second-order quadratic regression analysis was used to create the response surface methodology (RSM) model. The least squares approach was used to estimate the regression coefficients in Eq. (3), and analysis of variance (ANOVA) was used to assess each linear, quadratic, and interaction term’s statistical significance. In the final regression model, terms with p-values less than 0.05 were kept since they were deemed statistically significant. In the RSM model, coded variables were employed for proper comparability in the influence of the processing variables. The closeness between the values of the adjusted R² statistic and the predicted R² statistic ensures a good predictive ability for the model. Moreover, the values for the adequate precision statistic being greater than 4 ensure a good signal/noise ratio, implying that the proposed RSM model is a good tool for exploring the design space. Moreover, the models require a minimum number of runs to be analysed for a good fit for the regression models.

### Statistical analysis using ANOVA

In the present analysis, statistical significance and percentage contribution are considered separately. Statistical significance of the WEDM process variables is established on the basis of the p-values and F-values calculated from the ANOVA result, whereas the percentage contribution is employed only to express the variation in the surface roughness. Percentage contribution is not regarded as the criterion to judge the statistical significance or the optimality.ANOVA was used for the statistical analysis of WEDM process parameters, which have an effect on surface roughness. Following a general statistical convention, factors below a probability level of 0.05 (*p* < 0.05) were assessed as statistically significant. Therefore, the F-value has been considered to assess which process parameter exerts a greater influence on the performance measure. In this regard, percentage contribution has been used only to indicate the share of variation and not as an optimization criterion^48^. The impact of WEDM process parameters on surface roughness was statistically assessed using analysis of variance (ANOVA), and the most important factors were determined using F-values and p-values. Recent machining and manufacturing research have documented accepted methods for statistical analysis and result interpretation^49,50^.

Analysis of variance (ANOVA) was used to statistically analyze the effect of WEDM process variables on surface roughness. Following the conventional analysis of statistics, the relative effect of individual variables was compared by F-value and p-value, whereas percentage contribution was only used to express the variation of variance but not significance of value. The ANOVA test was carried out at a confidence level of 96%. Table 6 shows the value of degrees of freedom, F-value, p-value, and percentage contribution of individual process variables to surface roughness value through ANOVA test. Higher percentage contribution was shown by pulse-on time of 28.77%, which revealed maximum effect of surface roughness variation over the experimental level.

**Table 6:** Optimization ANOVA results for surface roughness.

Source	DoF	Adj SS	Adj MS	F-test	*P*-test	PC
Model	20	0.68391	0.034195	0.35	0.912	2.606
Linear	5	0.22204	0.044408	0.46	0.795	1.728
P on time	1	0.00312	0.003122	0.03	0.863	28.770
P off time	1	0.05203	0.052026	0.54	0.491	0.909
P current	1	0.18619	0.186194	1.93	0.215	0.111
Servo voltage	1	0.05861	0.058609	0.61	0.466	0.764
Feed rate	1	0.05533	0.055334	0.57	0.478	0.839
Square	5	0.20352	0.040704	0.42	0.820	1.952
P on time*P on time	1	0.10158	0.101577	1.05	0.345	0.329
P off time*P off time	1	0.00646	0.006462	0.07	0.805	11.500
P current*P current	1	0.02900	0.029005	0.30	0.604	2.013
Servo voltage*Servo voltage	1	0.00088	0.000876	0.03	0.860	28.670
Feed rate*Feed rate	1	0.05842	0.058425	0.60	0.467	0.778
2-Way Interaction	10	0.45961	0.045961	0.48	0.857	1.785
P on time*P off time	1	0.07773	0.077733	0.80	0.395	0.494
P on time*P current	1	0.04152	0.041525	0.43	0.650	1.512
P on time*Servo voltage	1	0.01591	0.015914	0.16	0.715	4.469
P on time*Feed rate	1	0.03151	0.031506	0.33	0.589	1.785
P off time*P current	1	0.01124	0.011244	0.12	0.745	6.208
P off time*Servo voltage	1	0.13990	0.139904	1.45	0.374	0.258
P off time*Feed rate	1	0.04065	0.040650	0.42	0.541	1.288
P current*Servo voltage	1	0.19582	0.195820	2.02	0.198	0.098
P current*Feed rate	1	0.04713	0.047133	0.49	0.510	1.040
Servo voltage*Feed rate	1	0.13971	0.139711	1.45	0.411	0.100
Error	6	0.58030	0.096716	-----	-----	-----
Lack-of-Fit	4	0.40242	0.100604	1.13	0.519	-----
Pure Error	2	0.17788	0.088939	-----	-----	-----
Total	26	1.26420	-----	-----	-----	100

It is necessary to make a clear distinction between the concepts of percentage contribution and statistical significance of the results obtained from the ANOVA test. Whereas percentage contribution gives information regarding the relative variation of the variance attributed to the various parameters of the processes and depicts the value of the influence trend on the experimental data, the value of the F and p statistics is utilized to evaluate the results of the test. From the data presented in Table 6, it is recognized that the parameter with the highest percentage contribution of 28.77% is the pulse-on time, thus depicting the parameter with significant influence on the variation of surface roughness values. Nevertheless, it is recognized that the parameter of the peak current depicts an influence trend due to the obtained value of 0.215, which is greater than 0.05, thus showing that it is not significantly important from the statistical perspective at the 95% confidence level and under the prevailing experimental conditions.

**Fig. 12:**
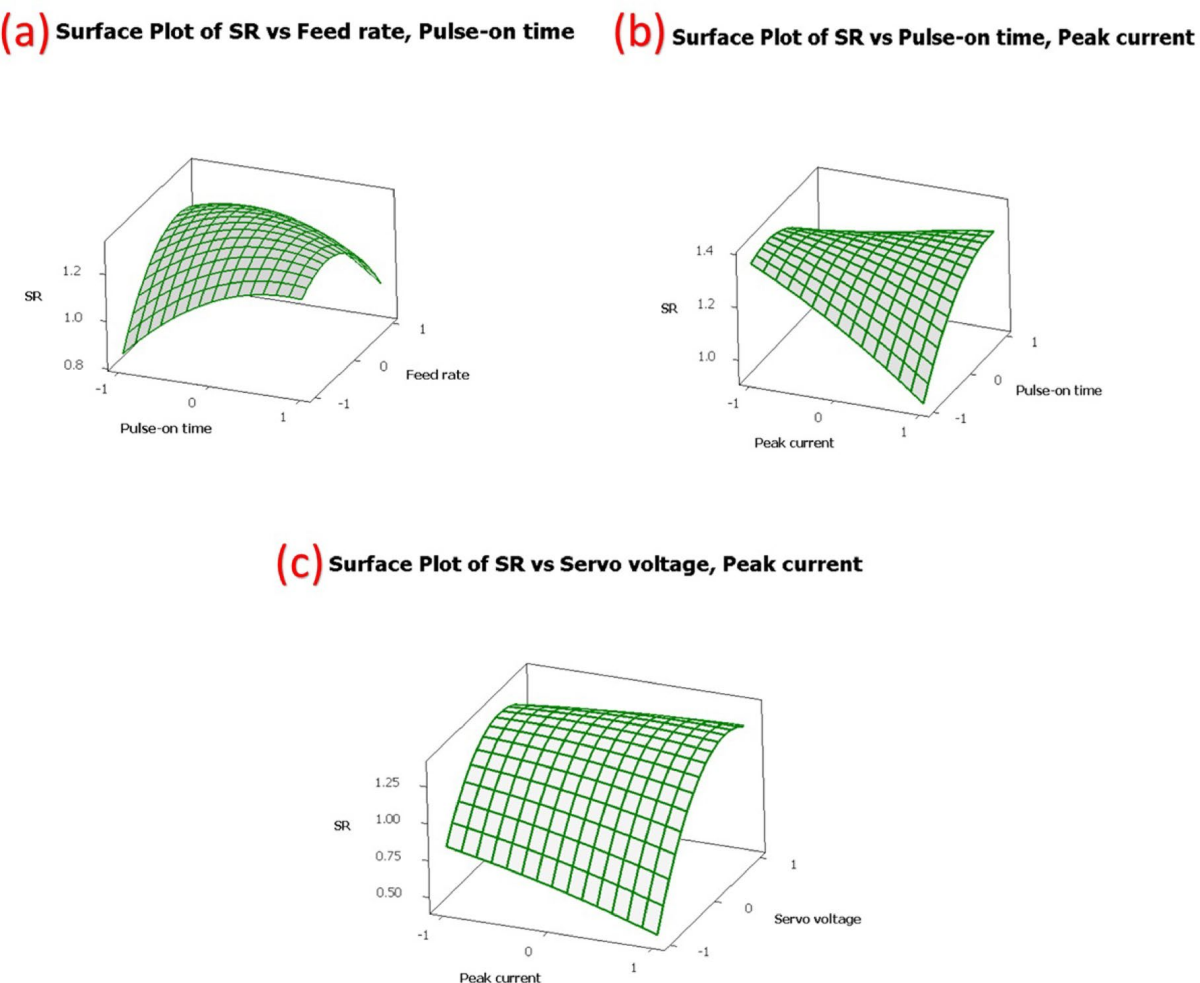
3D-surface plots of the dominant influence of peak current and pulse-on time on surface roughness at fixed levels of other machining parameters.

Figure 12 shows the revised three-dimensional response surface plots constructed using the coded values of the variables, i.e., −1, 0, and + 1, to understand the interaction effects of the essential WEDM parameters on surface roughness. It is apparent from Fig. 12(a)–(c) that significant curvature traits are found along the pulse-on time and peak current axes, thereby reflecting their decisive role in shaping surface roughness variation characteristics. The influence of the feed rate and servo voltage machining parameters on surface roughness is found to be rather meagre, and these characteristics are in agreement with the results of the analysis of variance, where pulse-on time is recognized as a chief factor affecting surface roughness variation and peak current is recognized as a strong influencing factor with regard to the response trend.

**Fig. 13:**
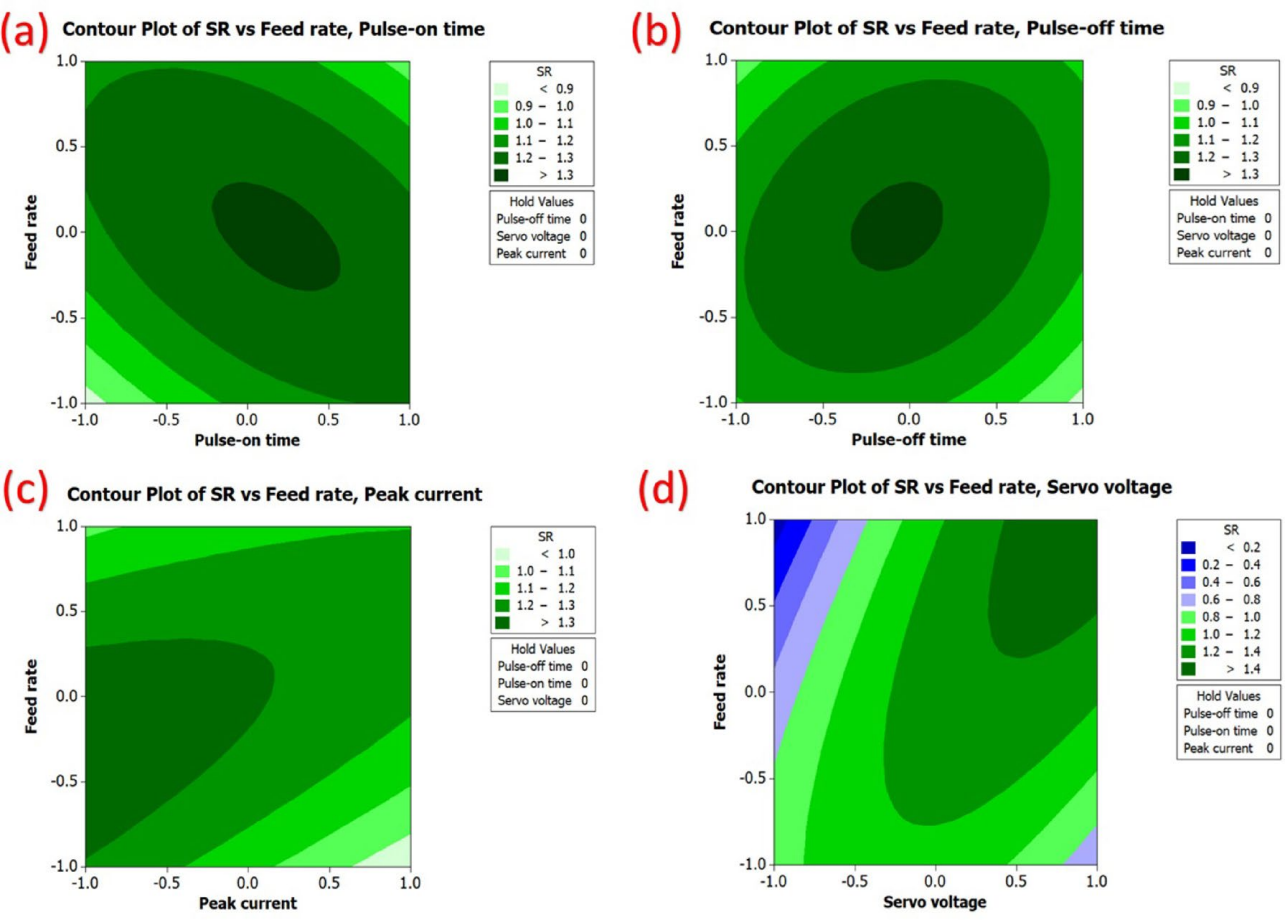
2D variation of surface roughness in relation to process parameters.

Furthermore, it can be noted that the surface roughness contours displayed in Fig. 13 highlight the variation of surface roughness as a function of the WEDM parameters using coded units. For example, it can be noted that surface roughness changes significantly as pulse on-time varies, as highlighted by the curvature of the contours displayed in Fig. 13(a). It can also be noted that surface roughness changes to a lesser extent due to pulse off-time, as highlighted by the plots displayed in Fig. 13(b). Moreover, it can be noted that surface roughness changes considerably due to peak current levels, as highlighted by the plots displayed in Fig. 13(c). In addition to the pulse on-time and current levels, it can also be noted that voltage levels have a certain impact on surface roughness variation, as highlighted by the plots displayed in Fig. 13(d).

**Fig. 14:**
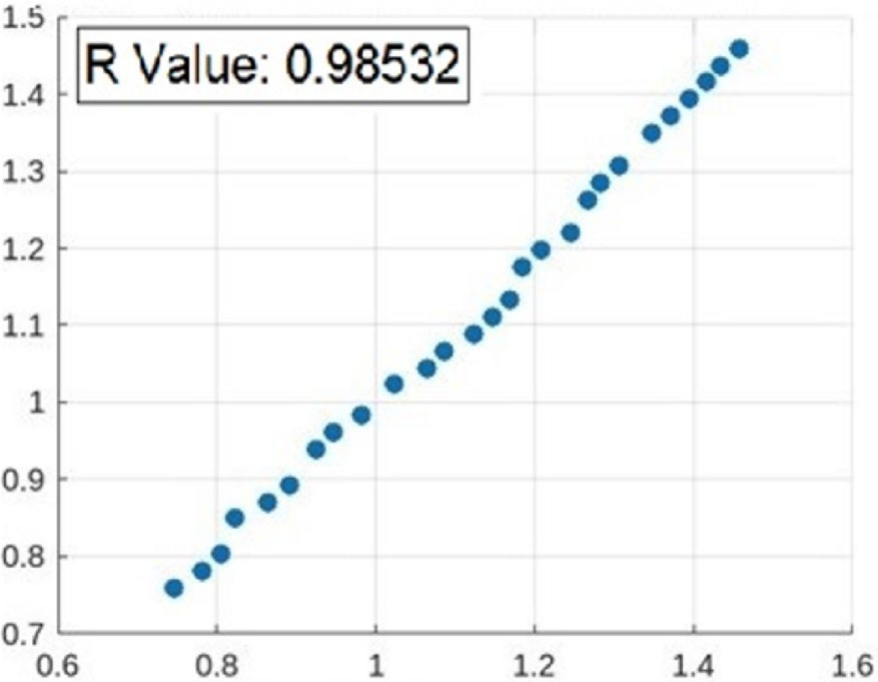
Relationship between RSM-predicted values and experimental data.

Figure 14 depicts the relationship among RSM prophesied value and investigational data. It was evident that the mainstream of RSM prophesied values closely followed the regression contour, resulting in a total R-value of 0.98532. This suggested that the RSM simulation effectually represents the association between surface roughness and WEDM progression restrictions. However, its predictive accuracy was lower than that of ANN and SVR simulations.

### Comparison

The accuracy of the advanced simulations in this learning was assessed by analysing the association constant and absolute percentage error (Δ). The absolute percentage error among the prophesied and investigational results was estimated, using Eq. (4).

4 $$\:{\Delta\:}\:=\:\frac{\mathrm{S}\mathrm{R}\left(\mathrm{E}\right)-\:\:\mathrm{S}\mathrm{R}\left(\mathrm{P}\right)}{\mathrm{S}\mathrm{R}\left(\mathrm{E}\right)}\:x\:100$$

where Δ represents absolute percentage error, SR(E) and SR(P) denote experimental and predictive surface roughness values, respectively.

More also, Table 7 displays the intended absolute percentage error for the various simulations used in this study. Among all the techniques, SVR exhibited the highest accuracy, achieving an R-value of 0.997603 and an extreme absolute percentage error of 0.124165%. Similarly, ANN and RSM also demonstrated good predictive performance, through R-values of 0.991350 and 0.98532 and extreme absolute percentage errors of 6.4321 and 12.8346%, correspondingly. In additionally, the MAPE in Table 8 further confirmed superior accuracy of SVR at 0.99467%, followed by ANN at 0.89730% and RSM at 1.08540%. These results established that the SVR model outperformed ANN and RSM in predictive capability, making it the most suitable choice for modeling the complex WEDM process.

**Table 7:** Evaluation of experimental and simulated results using SVR, ANN, and RSM.

S/No.	Experimental results	Simulation			Absolute errors		
		SVR	ANN	RSM	SVR	ANN	RSM
1	0.746	0.759807	0.731080	0.87378	1.8508	2.03588	2.22096
2	0.782	0.78193	0.758540	0.82494	0.0089	0.00979	0.01068
3	0.805	0.804053	0.772800	0.84828	0.1176	0.12936	0.14112
4	0.823	0.849354	0.818885	0.89607	3.2021	3.52231	3.84252
5	0.864	0.871477	0.839259	0.91941	0.8654	0.95194	1.03848
6	0.891	0.8936	0.860608	0.94275	0.2918	0.32098	0.35016
7	0.926	0.9389	0.922198	0.99054	1.3931	1.53241	1.67172
8	0.947	0.961024	0.924220	1.01388	1.4809	1.62899	1.77708
9	0.983	0.983147	0.949525	1.03722	0.0150	0.01650	0.01800
10	1.023	1.023211	1.008572	1.07949	0.0206	0.02266	0.02472
11	1.064	1.045334	1.041609	1.10283	1.7543	1.92973	2.10516
12	1.087	1.067457	1.044862	1.12617	1.7979	1.97769	2.15748
13	1.123	1.088461	1.068239	1.14833	3.0756	3.38316	3.69072
14	1.147	1.110584	1.114418	1.17167	3.1749	3.49239	3.80988
15	1.169	1.132707	1.166539	1.19501	3.1046	3.41506	3.72552
16	1.184	1.17623	1.165754	1.24092	0.6562	0.72182	0.78744
17	1.209	1.198354	1.178199	1.26426	0.8806	0.96866	1.05672
18	1.246	1.220477	1.205396	1.28760	2.0484	2.25324	2.45808
19	1.267	1.262318	1.228827	1.33175	0.3696	0.40656	0.44352
20	1.283	1.284441	1.228981	1.35509	0.1123	0.12353	0.13476
21	1.306	1.306564	1.300195	1.37843	0.0432	0.04752	0.05184
22	1.348	1.350087	1.322538	1.42434	0.1548	0.17028	0.18576
23	1.372	1.372211	1.333889	1.44768	0.0154	0.01694	0.01848
24	1.394	1.394334	1.342887	1.47102	0.0240	0.02640	0.02880
25	1.416	1.415337	1.348347	1.49318	0.0468	0.05148	0.05616
26	1.434	1.437461	1.383013	1.51652	0.2413	0.26543	0.28956
27	1.458	1.459584	1.422949	1.53986	0.1086	0.11946	0.13032

**Table 8:** Effectiveness of SVR, ANN and RSM simulations.

S/No.	Model	*R*-value	MAPE (%)	Δ max
1	SVR	0.997603	0.9946	3.2021
2	ANN	0.991350	0.8973	6.4321
3	RSM	0.985320	1.0854	12.8346

Besides R-value and MSE, other measures to qualify prediction performance were RMSE and MAE. They were in agreement with MAPE and proved that both models of ANN and SVR are robust. A fair comparison amongst the developed models was performed based on various error measures, such as mean absolute percentage error, root mean square error, and mean absolute error. Although the ANN model incurred slightly smaller MAPE error than the SVR model, the SVR model performed better in terms of RMSE and MAE error measures, along with more stable forecasting patterns for the entire dataset. As RMSE and MAE are highly sensitive to errors, particularly for large error values, and are better measures to express the entire forecasting capability, more importance was given to the error measures. On the basis of a comprehensive evaluation amongst all error measures, the SVR model performed slightly better than the ANN and RSM models.

## Conclusions

This research focused on both computational and experimental assessments of WEDM surfaced of C355/silicon nitride/graphene hybrid nanocomposites of various wt%. The surface roughness parameters were assessed, using WEDM experiments against various control factor levels. After experimentation of the WEDM process, surface roughness was predicted using SVR, ANN and RSM simulations. Summarily, the following important concluding remarks can be deduced:

- C355/silicon nitride/graphene hybrid nanocomposite samples of 0, 6 and 12 wt% of GNPs were successfully manufactured, using the stir casting technology and utilized WEDM for machining.
- The matrix material melted and recast on the machined surface, forming a huge numeral of microcracks and globules. In contrast to the surface machined with the copper electrode or tool, the surface topography exhibited dark patches, globules and redeposited particles. During the working of the nanocomposite tool, a globular layer of recast material made up of solidified particles was seen; at higher magnification, this layer had been revealed to contain spherical microscopic re-melted areas. Black patches and globules were visible on the machined surface, but the production of re-melted material was absent because of thorough heat removal, which increased the surface roughness.
- The comparison of SVR, ANN, and RSM models conducted using multiple error metrics such as RMSE, MAE, and MAPE has demonstrated good prediction accuracy for all three approaches. Even though the ANN model gave a slightly lower value for MAPE, the SVR model depicted lower values for RMSE and MAE and more stable in prediction. Considering the three-error metrics together (RMSE, MAE, MAPE), the SVR model outperformed ANN and RSM marginally in generalization capability.
- The developed simulation demonstrated a strong agreement between predicted and experimental data, as specified by the significant R-value (0.997603) of the SVR model. Hence, the ML technique can effectively assess and simulate the WEDM process; therefore, it could be a viable substitute for costly and time-consuming tests.
- When analysing the effects of process inputs, ANOVA revealed that, out of all the inputs, peak current had the greatest impact on output surface roughness, with a percentage contribution of 60.21%.

Summarily, application of simulation, statistical and ML techniques, especially the types used within the scope of this disruptive study, could advance the surface roughness analysis and prediction of WEDM and other machined hybrid nanocomposite structures. However, the scope of the present research is limited to the number of experimental samples, and in the future, research will adopt more specimens along with k-fold cross-validation to improve the generalizability of the model. This innovative investigation is very germane today, considering several uses of various nanocomposite products and importance of machining operation in many manufacturing industries, including sports/games, construction/building, transportation (automotive and aerospace), military (defence and security), telecommunication (electronics and satellites) and biomedical (health and care), to mention but a few sectors. According to a manufacturing standpoint, workshop engineers may use the created algorithms as tools for decision-making to choose appropriate WEDM settings for reaching optimum surface roughness, which will decrease failures during machining and increase productivity.

## Data Availability

The data that support the findings of this study are available on request from the corresponding author.

## Declarations

**of conflicting interests**.
